# Reproducibility of Fluorescent Expression from Engineered Biological Constructs in *E. coli*

**DOI:** 10.1371/journal.pone.0150182

**Published:** 2016-03-03

**Authors:** Jacob Beal, Traci Haddock-Angelli, Markus Gershater, Kim de Mora, Meagan Lizarazo, Jim Hollenhorst, Randy Rettberg, iGEM Interlab Study Contributors

**Affiliations:** 1 Raytheon BBN Technologies, Cambridge, MA, United States of America; 2 iGEM Foundation, Cambridge, MA, United States of America; 3 Synthace, London, United Kingdom; 4 Agilent, Santa Clara, CA, United States of America; Cardiff University, UNITED KINGDOM

## Abstract

We present results of the first large-scale interlaboratory study carried out in synthetic biology, as part of the 2014 and 2015 International Genetically Engineered Machine (iGEM) competitions. Participants at 88 institutions around the world measured fluorescence from three engineered constitutive constructs in *E. coli*. Few participants were able to measure absolute fluorescence, so data was analyzed in terms of ratios. Precision was strongly related to fluorescent strength, ranging from 1.54-fold standard deviation for the ratio between strong promoters to 5.75-fold for the ratio between the strongest and weakest promoter, and while host strain did not affect expression ratios, choice of instrument did. This result shows that high quantitative precision and reproducibility of results is possible, while at the same time indicating areas needing improved laboratory practices.

## Introduction

Rapid improvements in our ability to both understand and genetically engineer biological organisms offer the potential for revolutionary applications for medicine, manufacturing, agriculture, and the environment [1–5]. A major barrier to transition from principle to practice, however, is the frequent sensitivity of biological systems to small changes in their cellular or environmental context [6]. This makes it difficult to reproduce or build on prior results in the lab, let alone to ensure desirable behavior in a deployed application, and may play a part in significant concerns that have been raised with respect to the state of published biomedical literature [7–10].

Practitioners of synthetic biology face particularly strong challenges in this area, because the engineering approaches applied in this area are often particularly demanding of quantitative precision in models and measurements. At the same time, synthetic biology offers a unique opportunity for improving our understanding of biological systems, through insertion of artificial systems intended to operate relatively independently from the evolved systems of their host or else to apply precise interventions to that host. Due to their engineered nature, these systems are likely to be more tractable to study than natural systems, as well as to provide leverage for the study of natural systems.

An important step toward addressing these issues is to quantify the degree of variability exhibited by engineered genetic constructs across different laboratories. Toward this end, we present the results of the first large-scale interlaboratory study of reproducibility in synthetic biology, carried out by students at 88 institutions around the world as part of the 2014 and 2015 International Genetically Engineered Machine (iGEM) competitions. This study focused on one of the most widely used tools in genetic engineering: constitutive expression of fluorescent protein from an engineered plasmid (although there are a number of well-known disadvantages to assays based on fluorescent proteins, such as oxygen dependence and folding times, these techniques are readily accessible, widely used, and still one of the best and easiest ways to study many biological phenomena). In particular, each participating team measured three engineered constitutive constructs in *E. coli* grown under standardized conditions following a particular protocol. Previously, a small-scale interlaboratory study with similar design demonstrated that it is possible for several laboratories to obtain relative measurements accurate to within a 2-fold range using flow cytometry [11]. Our new study enhances these results with a much wider range of participants and instruments, as well as identifying likely sources of variation and key challenges to be addressed.

In particular, we find that strong fluorescent expression can be measured with a remarkable degree of precision—to a maximum of 1.54-fold standard deviation. Precision degrades significantly, however, for constructs with weaker expression. Surprisingly, we find no significant correlation between strain and observed behavior, suggesting that the constructs measured are not overly sensitive to host context, while choice of instrument does appear to affect results. These results are promising, in that they show that engineered genetic constructs can exhibit a high degree of consistency in behavior even in the face of significant variations in context. At the same time, the limits encountered in this study highlight the need for adoption of calibrated measurements producing standardized units (e.g., [12, 13]) and for protocol approaches that can reduce the impact of “cultural art” in laboratory methods (i.e., the undocumented or undocumentable differences in how an apparently identifical measurement is executed differently between two individuals or laboratories, and in how the data is handled to turn “raw” observations into reported values), which can be reduced by methods such as [14] and [15]. Finally, this study also demonstrates how critical issues in science and reproducibility can be addressed through focused undergraduate research challenges and “citizen science” involving students around the world.

## Materials and Methods

**Fig 1:**
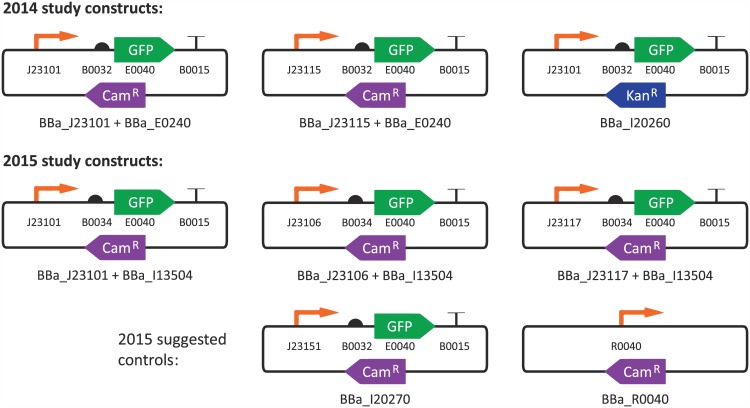
Constitutive fluorescence constructs measured in the 2014 and 2015 iGEM Interlab Studies.

The aim of this study is to establish a broadly relevant baseline for precision and replicability in engineered biological systems. As such, we selected the following experimental conditions as being both readily accessible to laboratories at varying levels of sophistication world-wide, and also representative of much work in biological engineering:

- **Host organism:** *E. coli* (preferably a DH5-alpha strain)
- **Engineered construct:** constitutive expression of green fluorescent protein at three levels (“strong”, “medium”, and “weak”), driven by constitutive promoters from the Anderson promoter collection [16] (the same collection drawn from in [11]). Constructs are shown in Fig 1, with additional details provided in S1 File.
- **Molecular cloning:** BioBricks Assembly [17] was recommended for building constructs, but not required; some teams substituted DNA synthesis or other assembly strategies.
- **Culture conditions:** 16–18 hours of growth in LB broth plus appropriate antibiotic, at 37°C and shaking at 300 rpm.
- **Replication:** triplicate
- **Measurement:** green fluorescence measured as best capable, in absolute SI units if possible.

In the 2014 iGEM Interlab Study, the protocol was intentionally left at this vague level of specification, for two reasons. The first goal was to determine what types of equipment were commonly accessible at participating institutions worldwide. The second goal was to determine how much variation was common in protocol execution and reporting, for both culturing and measurement.

Following analysis of the 2014 study, the protocol was adjusted with the aim of improving precision. In particular, the following key changes were made for the 2015 iGEM Study:

- The set of constructs measured was changed to all use the same plasmid backbone (thus eliminating differences in growth and expression due to the use of different antibiotics) and to have a greater range of expression between constructs.
- A set of defined positive and negative controls were added.
- Standardized protocols were specified for construction, culturing, and measurement, along with checklists and forms for participating teams to fill out as they executed these protocols. These were split across two forms, attached as S2 and S3 Files.
- When possible, teams were encouraged to provide data in both biological and technical triplicate (for a total of nine replicates). Standardized reporting of individual sample measurements also allowed elimination of anomalous samples: in cases where one sample differs from all the rest by at least 5-fold or one set of replicates differs by at least 3-fold (and values are not very close to zero), those samples were not used for analysis.

Both studies were advertised to all teams participating in iGEM through an information page on the iGEM website, as well as social media and iGEM newsletters. The information pages for the 2014 and 2015 studies are attached as S4 and S5 Files, respectively. Beginning from the initial advertisement, teams had several months in which to indicate that they were volunteering to collect data for the study, to execute the specified protocols and collect fluorescence data, and finally to return protocol records and fluorescence data via email or web forms. Finally, we analyzed the returned data and protocols and presented preliminary results and recognition of the contributing teams at each year’s iGEM Giant Jamboree.

For the 2014 study, 45 teams participated, and of these 36 teams were ultimately able to contribute data for inclusion in the study. For the 2015 study, 85 teams participated, and of these 67 teams were ultimately able to contribute data for inclusion in the study. There were 18 teams that participated in both studies, of which 15 contributed data to both studies and 2 contributed data to only one of the studies. The results presented in this paper thus represent experiments conducted at 88 institutions worldwide. These institutions are widely distributed across countries and continents: contributing teams hail from 27 different countries, with 42 teams from Europe, 19 teams from Asia, 19 teams from North America, 7 teams from Central and South America, and 1 team from Oceania. The vast majority of participants were undergraduate students, but many teams also included or were comprised of graduate students or high-school students. All members designated by a team as deserving co-authorship for their contributions are recognized as consortium authors in the Acknowledgments section.

## Results

For the 2014 study, almost all contributing teams produced one usable set of measurements. Of the 36 teams contributing data, five teams measured samples with two instruments, but issues in data reporting means that all but two of those dual data sets had only one readily usable set of data. Accordingly, we analyze using only the best documented dataset for each team, a total of 36 datasets.

For the 2015 study, a number of teams decided to collect more than one dataset, using multiple instruments and/or multiple strains: all told, the 67 contributing teams produced a total of 95 datasets. This presents an additional opportunity for intra-team data analysis, which will be explored below in our examination of sources of variability.

All values in these datasets are reported in S1 and S2 Tables. In addition, data from the 2015 study protocol and measurement forms is included as S3 and S4 Tables.

### Absolute vs. Relative Units

First and foremost in our analysis, we observed that almost none of the datasets include data in directly comparable SI units. Only two datasets from the 2014 study are reported in absolute units, and the units obtained for these datasets are not comparable. In the 2015 study, twelve teams reported at least one dataset in absolute units, generally calibrated to a standard fluorophore, most frequently fluorescein. Two groups of these datasets are directly comparable: two datasets are reported in MEFL (molecules of equivalent fluorescein), and four datasets are reported in ng/mL fluorescein.

In both of these groups of data, however, the ratios between constructs within the datasets are quite similar, while there are two or more magnitudes of difference across the absolute numbers reported. It thus appears likely that calibration techniques are being applied inconsistently. Highly inconsistent calibration is effectively no better than simply using arbitrary units to begin with. Thus, as is frequently the case in current biological research, we cannot directly compare measurements from one lab to another.

We can still, however, consider the precision and reproducibility of relative fluorescent expression. Accordingly, the remainder of our analysis will use normalized fluorescence, comparing the ratio of the constructs most similar between the two years (Strong14 and Strong15) to each other promoter measured. This gives us the following five sets of ratio data to analyze:

- 2014: Strong14/Medium14, Strong14/Weak14
- 2015: Strong15/Medium15, Strong15/Positive15, Strong15/Weak15

The exact construct for each device is noted in Fig 1 and S1 File. Note that for the 2015 study we include ratios with the suggested positive control, as teams used this construct for 75 data sets, producing a significant body of data to analyze. Negative controls, however, were highly variable in both identity and data handling, including many zero, negative, and near-zero numbers. This renders them a poor target for ratiometric analysis, and we thus do not include them in our analysis.

The remainder of our analysis focuses on the five expression ratios that we have identified here.

### Replicability and Precision

In evaluating precision and replicability, we began with a hypothesis about the sources of variation that are likely to be encountered. In particular, we hypothesized that observed variation can be viewed as a mixture of three main sources of variation:

- **Mistakes and Failures:** The most extreme variations will likely be driven by gross protocol failures, such as contamination of samples, incorrect assembly of constructs, mixing up samples, or mistakes in data interpretation and entry. The occurrence of such failures is often best addressed by replication and by improvement of controls and procedures, such that mistakes and failures can be more easily detected and an affected experiment repeated.
- **Communication-Based Variability:** Some variations are driven by differences in how various people in various laboratories interpret a given experimental protocol. These types of variation can often be addressed by standardization and training, and by more precise communication of protocols.
- **Systemic Variability:** Some variation is due to the nature of the system under observation, e.g., inherent variability of biological organisms, precision limits of instruments, variation in reagents, environmental differences (e.g., laboratory altitude). This type of variation is often the most difficult to address, as it is likely to involve fundamental, technological, or economic limits.

Single-cell fluorescence levels tend to vary following an approximately log-normal distribution (see e.g., [12, 18–21]). As such, we hypothesize that the ratios in the data-set will also vary following a log-normal distribution, plus some outliers caused by mistakes and failures (Note that it is for this reason that we report variability as *x*-fold rather than ±*x*: a plus/minus range on a log-scale translates to a multiply/divide range in the standand linear scale. Thus, for example, one 2-fold standard deviation around a mean of 10 is a range of 5 to 20, and two standard deviations is a range from 2.5 to 40).

**Fig 2:**
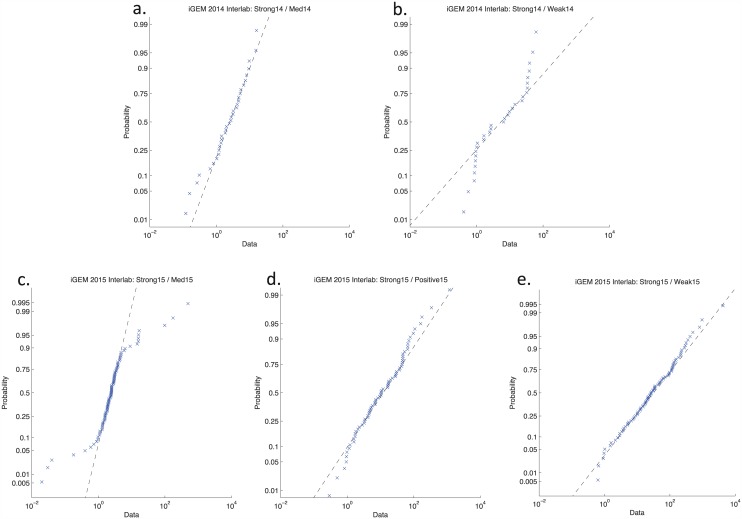
Log-Normal distribution diagrams for fluorescence expression ratios. The mostly linear structure indicates that variation in expression does generally conform closely to log-normal distribution, with more vertical clustering indicating tighter distributions. Data points significantly off the line will be considered outliers and not used in further analysis of these distributions.

To evaluate this hypothesis, we constructed log-normal distribution diagrams for each fluorescence expression ratio, in which sorted data is plotted against the expected probability distribution (Fig 2). The mostly linear structure of these plots indicates that variation in expression does generally conform closely to log-normal distribution, with more vertical clustering indicating tighter distributions.

Data points significantly off of the linear distribution will be considered outliers: Table 1 gives the number of points that we are interpreting as outliers above and below the log-normal distribution of each data set. These we attribute to mistakes and failures and will not use in further analysis aimed at understanding the relationship between precision of communication and system variability. In total, 17% of all data points are outliers, and the rate of outliers in the 2015 data set is lower than in the 2014 data set. We hypothesize that the decreased rate of outliers is due to the use of more detailed protocol instructions and checklists, but there is not enough data to conclude that this difference is necessarily significant.

**Table 1:** Number of data points total for each ratio, along with number and fraction of data points that we interpret as outliers (i.e., not conforming to the log-normal distribution hypothesis).

Expression Ratio	Samples	Non-Outliers	Outliers		
			High	Low	Fraction
Strong14/Medium14	34	28	1	5	0.18
Strong14/Weak14	32	18	7	7	0.43
Strong15/Medium15	94	77	9	8	0.18
Strong15/Positive15	71	55	9	7	0.22
Strong15/Weak15	90	89	0	1	0.01
Total	321	267	26	28	0.17

It is also possible that some outliers may be due to sequence variations induced by different assembly methods, since assembly scars are known to have a significant effect on promoter expression [22]. This appears to be unlikely, however: in 2015, when teams were required to report their assembly method, 10 data sets were produced with methods other than BioBricks. These account for only three Strong15/Medium14, three Strong15/Positive15, and no Strong15/Weak15 outliers, a proportion in line with the overall fraction of outliers.

**Fig 3:**
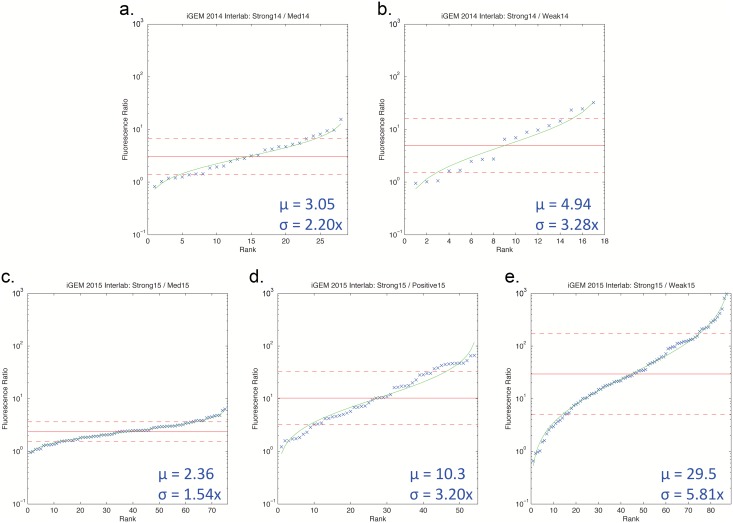
Rank-sorted data for each ratio, omitting outliers. Blue points are observed ratios, red lines show geometric mean (solid) and ±1 std.dev. (dashed), and green line shows log-normal distribution fit.

**Fig 4:**
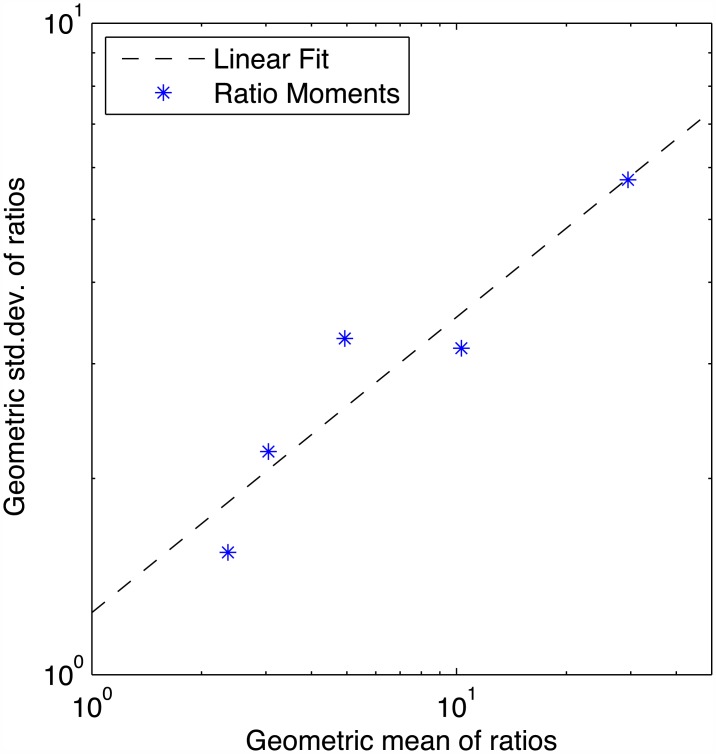
Relationship between mean ratio and precision. The lower another promoter’s expression is in relation to the strong construct, the higher the variation in measurement: standard deviation grows approximately in proportion to the square root of the mean ratio.

Turning to the non-outlier data, Fig 3 shows a plot of rank-sorted data against a log-normal distribution model, along with the mean (*μ*) and standard deviation (*σ*) for each distribution. Surprisingly, there is a great degree of variation in the precision of measurements. The lower a promoter’s expression is in relation to the Strong14 or Strong15 construct, the higher the variation in measurement. Fig 4 plots mean against standard deviation, showing that standard deviation grows approximately in proportion to the square root of the mean ratio. This might indicate either that measurement is less precise for weak fluorescence or that error grows as the two values grow farther apart. We can test which is more likely by considering the other four ratios: Medium14/Weak14, Medium15/Positive15, Medium15/Weak15, and Positive15/Weak15. S1 and S2 Figs show the results, which indicate that error appears to be controlled more strongly by the denominator, suggesting that the problem is in fact decreased precision in quantifying weak fluorescence. This conclusion is further supported by our analysis of instrument-linked variation in the next section.

### Sources of Imprecision

To begin investigation of possible sources of variation, we identified the two largest and most systematic differences in protocol from team to team: the strain of *E. coli* used for culturing, and the instrument used to measure behavior. For this analysis, we coded strains into four categories, as shown in Table 2, and instruments into five categories, as shown in Table 3.

**Table 2:** Strains reported in 2014 and 2015 iGEM Interlab Studies.

Strain	2014	2015
DH5-alpha	16	50
TOP10	7	16
BL21	2	9
Other	6	20
Not reported	5	—

**Table 3:** Instruments reported in 2014 and 2015 iGEM Interlab Studies.

Instrument	2014	2015
Plate Reader	23	56
Flow Cytometer	6	25
Microscope	3	7
Other Spectrofluorimeter	2	5
Other	2	2

**Fig 5:**
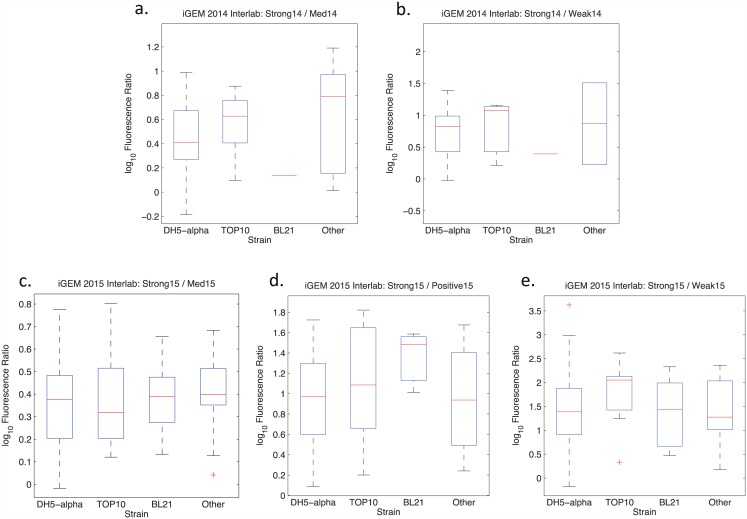
Distributions of data partitioned by *E. coli* strain for each ratio, omitting outliers. Note that the BL21 subset in 2014 has only a single non-outlier data point and may thus be effectively ignored.

**Fig 6:**
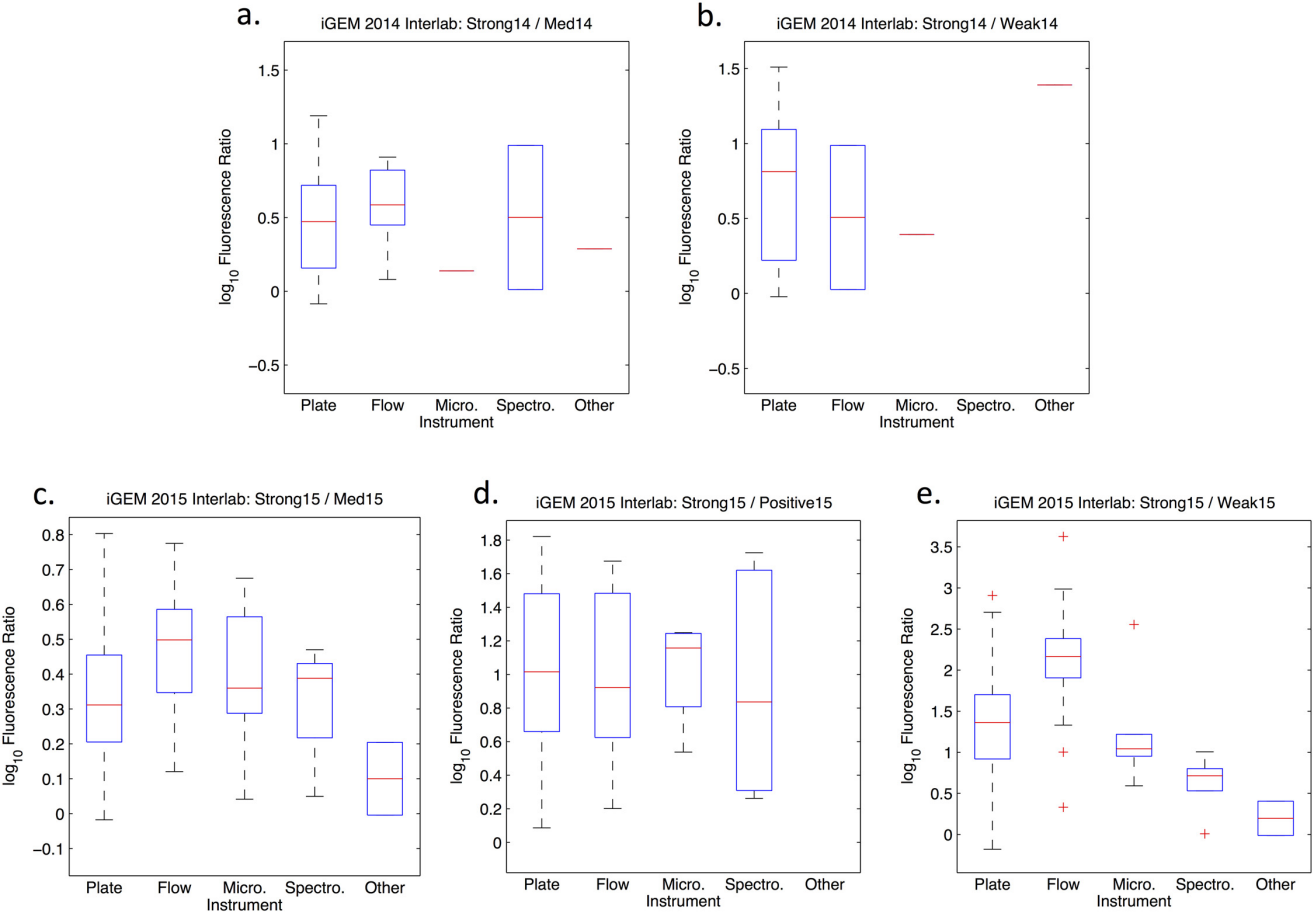
Distributions of data partitioned by measuring instrument for each ratio, omitting outliers. Note that some subsets have only one or zero non-outlier data points and may thus be effectively ignored.

The results, shown in Figs 5 and 6, were unexpected. While we had expected to see significant variation by strain, the distributions were for the most part quite consistent from strain to strain. Between classes of instruments, however, there appears to be more variability. Most notably, there is more than a 6-fold difference between the mean values returned by flow cytometry and by other methods for the Strong15/Weak15 ratio. Such instrument-linked variability might be due to the instruments themselves, but could also be due to other causes, such as differences in how measurement and analysis is carried out on different instruments by different people.

**Fig 7:**
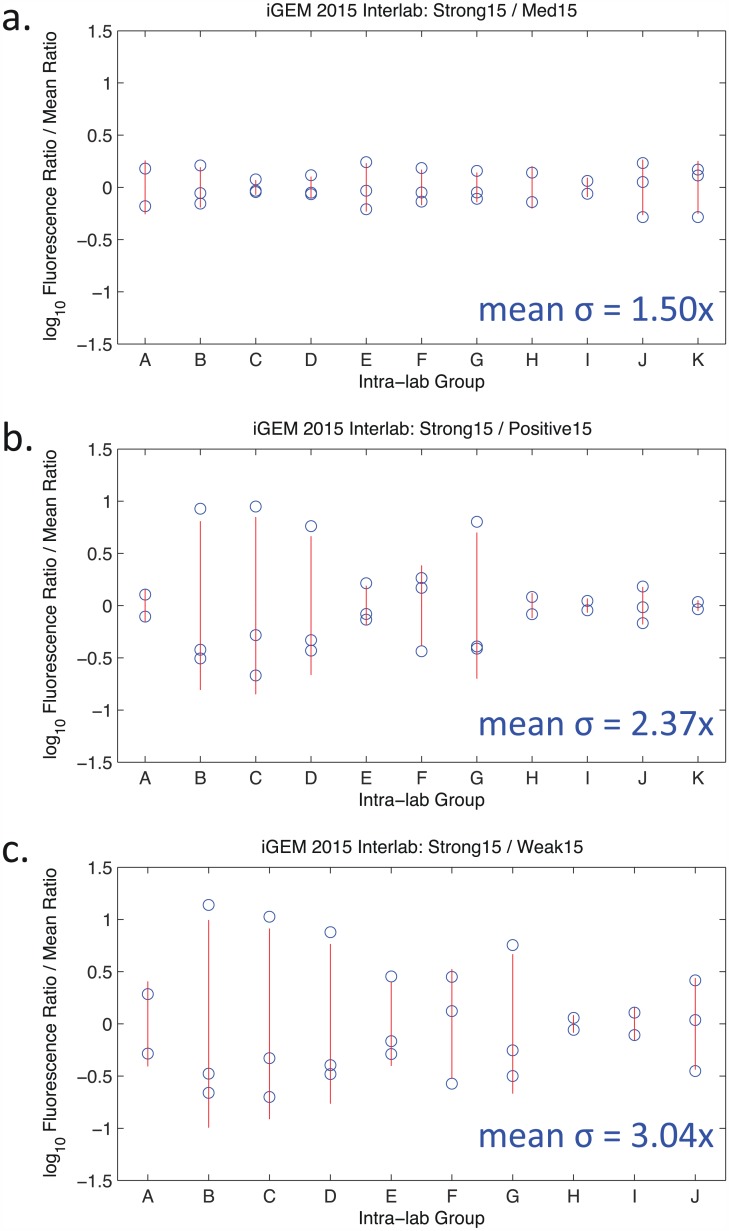
Instrument-to-instrument variation within a single team. Each column represents a set of replicates measured by the same laboratory on different instruments. Blue circles are the ratios for individual instruments, normalized by mean ratio; the red line spans ±1 std.dev.

To further investigate the possibility that instrument-linked variation is a source of significant error, we take advantage of the fact that a number of the contributing teams for the 2015 study contributed multiple data sets per sample by measuring the same samples with different instruments. For the ratios with Medium15 and Positive15, there are 11 available data-sets, and for Weak15 there are 10 available data-sets. Most of these have three measurements, two of which are a plate reader and a flow cytometer. If instrument-linked variation is a driving force in imprecision, then the intra-team variation should be similar in scale to the team-to-team variation reported in Fig 3. This is in fact the case, as shown in Fig 7. Most importantly, note that instrument-linked variation is larger for ratios comparing with lower expression samples, just as it is for variation across data sets overall.

As a cross-check, we also compute the variation between replicates. This variation is much tighter: the geometric mean of geometric standard deviations across ratios for individual samples is only 1.24-fold, meaning that the fundamental measurement variation of individual instruments can account for at most approximately half of the tightest observed distribution.

Together, all of these various pieces of evidence support the hypothesis that measurement precision is poor for weak fluorescence, and that this is largely due to differences in how measurement and analysis is carried out by various laboratories, rather than systemic variability in either the biological organisms or the instruments themselves.

## Discussion

Our results reveal a mixed picture of reproducibility and precision for engineered expression of fluorescence. On the positive side, the fraction of outliers observed is not too great, and the distributions of results conforms overall quite nicely with the prediction of log-normal distribution. Furthermore, we see that relative expression can be quite tightly quantified for pairs of strong fluorescent proteins.

On the negative side, however, the lack of standardized absolute units limits analysis to comparison of relative levels, which are much less useful than absolute units. Critically, we believe that the difficulty of performing “quality control” on a data set is a key contributor to both the number of outliers and to the variation between non-outlier measurements, since mistakes and failures cannot be readily excluded. Absolute unit measurements can allow control samples to be used for more stringent quality control of experiments by direct comparison of control measurements against expected standard values, thereby allowing detection of experimental or analytic problems and potentially greatly improving data quality (e.g., as described in [23]).

The difficulty of quantifying larger ratios is also quite concerning, as is the significant variation of values from instrument to instrument, even for the same samples within a single laboratory. We hypothesize that this phenomenon indicates general difficulties in quantifying weaker levels of fluorescent expression. It is not surprising that weak signals are more difficult to quantify than strong signals. What is surprising is that the dynamic range of effective quantification appears to be so narrow, since fluorescence is routinely quantified across much wider dynamic ranges than the mean strong/weak ratio observed in this study. Our suspicion is that this is not fundamentally due to the instruments (though flow cytometers do typically have a wider dynamic range than the other instruments used), but rather due to differences in how various laboratories deal with background fluorescence and with data interpretation and analysis.

One possible criticism of this study is the predominance of undergraduate researchers. We argue, however, that undergraduate researchers are more representative of the actual culture and practice of a laboratory than is perhaps comfortable to admit. There is a good deal of similarity between undergraduate researchers and the situation of a new graduate student or even a postdoctoral researcher entering a laboratory and learning the particulars of its protocols, equipment, and practices. This may be further intensified by the interdisciplinary nature of the field, meaning that even senior researchers often come from highly diverse backgrounds.

Overall, we think that the results of this study are largely good news for synthetic biology and for any other biological research that makes use of engineered expression of fluorescent proteins. First, the Anderson collection promoters seem to be fairly stable in their performance between different strains of *E. coli*, meaning that they are indeed generally a good starting point for tuning of gene expression. Second, we have established a clear baseline for precision and reproducibility. Since this has been established with the aid of undergraduates at a wide variety of institutions around the world, we argue that it should be considered as strong minimum standard for accuracy and reproducibility of published work.

Paradoxically, the measurement problems highlighted in this study may also be good news. Is is not surprising that this study shows problems in precision and reproducibility: challenges with variation in behavior and difficulty in reproducing and building upon results are commonly voiced concerns within the field. What is surprising and significant, however, is that they appear to be tied closely to the use of instruments and/or analysis of data. The instruments themselves appear to be quite reliable, as indicated by the tight replicate-to-replicate distribution. If the instruments are reliable, yet the same sets of samples produce markedly different relative values on different instruments, then this indicates that the problem is likely to be in how people are assaying cells with their instruments and the how they are analyzing the data produced by those instruments. This is likely to be compounded by differences in sensitivity and detection range: for example, flow cytometers typically have a wider range than plate readers, and thus a flow cytometer data set is likely to be less sensitive to how weak signals and background fluorescence are handled. In short, these simple biological systems appear to be much less variable than the ways in which we are studying them. This is good news because if the variability in precision and reproducibility was primarily systemic—i.e., due to the biological systems being studied, then it might be very difficult or even impossible to make significant improvements. Instead, however, much of the observed variability appears to be communication-based, i.e., closely tied to the ways in which people are studying those systems, and there are thus many good possibilities for rapid improvement in precision.

In particular, to address variability in fluorescent measurement, we recommend pursuit of three methods for increasing precision, listed here in order of ease of adoption, are:

- **Dissemination, training, and standardization around improved protocols for calibration of assays.** There are well-established protocols for calibration of flow cytometers to absolute units [12, 23–26], but these have not yet been widely adopted outside of the medical diagnostic community. Other calibration protocols exist for measurement of population fluorescence (e.g., [13, 27, 28]), but these also have not been effectively disseminated through the community.
- **Removing “craft” from protocol execution.** Much of the training that scientists receive in wet-lab protocols has an aspect of “apprenticeship” about it: important “craft” information is transmitted that is not formally written down in any location, and this makes it more difficult for protocols to be correctly replicated. A number of systems have been developed which make attempt to regularize the manner in which humans execute protocols (e.g., [14, 29]). These approaches still have lab work being done by humans, but apply techniques such as detailed checklists and computer monitoring of protocol execution to decrease the skill required and the variability of results produced by humans executing protocols.
- **Increased automation of protocols.** Variability in protocol execution can be reduced yet further by entirely or almost-entirely automating the execution of protocols. The two main approaches currently being developed in these areas are robotics (e.g., [30–33]) and microfluidics (e.g., [15, 34]). In the long run, this is where most of the field will likely go, just as automation has produced gains in productivity and precision in most other fields. At present, adopting protocol automation is impractical for most laboratories, as doing so generally requires a large investment of time and resources. Ongoing miniaturization and models for outsourcing to “cloud labs,” however, may soon make this technology much more widely accessibly.

Effective use of any of these approaches also requires deepening our understanding of both the systems we study and the means applied for studying them. We need to know which aspects of protocols are most sensitive, and which are susceptible to optimization and modification to fit circumstances, we need better controls and procedures for eliminating outliers, and we also need to extend and apply all of these principles beyond constitutive fluorescence in *E. coli* to a much wider range of organisms and engineered systems. Toward this end, we are making the dataset collected in this study available to the scientific community for deeper, multifactorial analysis, and intend to organize further interlaboratory studies putting our hypotheses to the test and extending their range of study.

## Supporting Information

S1 File DNA Constructs. DNA constructs for the 2014 and 2015 iGEM Interlab Studies. (PDF)

S2 File 2015 Study Protocol. Detailed protocol specification and reporting form provided for 2015 iGEM Interlab Study. (PDF)

S3 File 2015 Study Measurement Reporting. Detailed measurement reporting form provided for 2015 iGEM Interlab Study. (PDF)

S4 File 2014 Study Information Page. Information and instructions provided for 2014 iGEM Interlab Study. Document provided is a snapshot of the page as of October 4th, 2015, which includes information presented on participation and the preliminary results of the study.
(PDF)

S5 File 2015 Study Information Page. Information and instructions provided for 2015 iGEM Interlab Study. Document provided is a snapshot of the page as of October 4th, 2015.
(PDF)

S1 Table 2014 Summary Data. Datasets analyzed for the 2014 iGEM Interlab Study. (CSV)

S2 Table 2015 Summary Data. Datasets analyzed for the 2015 iGEM Interlab Study. (CSV)

S3 Table 2015 Protocol Form Responses. Response information from the protocol specification and reporting form for the 2015 iGEM measurement study. Some teams returned their forms (or portions thereof) manually for a number of reasons, most frequently due to Internet censorship in their home countries; entries from these teams are not included in the attached form. Only technical entries for the form are included, and team names have been replaced by numbers corresponding to the numbers in the summary data table.
(CSV)

S4 Table 2015 Measurement Form Responses. Response information from the measurement reporting form for the 2015 iGEM measurement study. Some teams returned their forms (or portions thereof) manually for a number of reasons, most frequently due to Internet censorship in their home countries; entries from these teams are not included in the attached form. Only technical entries for the form are included, and team names have been replaced by numbers corresponding to the numbers in the summary data table.
(CSV)

S1 Fig Additional Log-Normal Distribution Graphs. Log-normal distribution graphs for additional ratios. Graphs are attached for:
Medium14/Weak14Medium15/Positive15Medium15/Weak15Positive15/Weak15
(PDF)

S2 Fig Additional Rank-Sorted Graphs. Rank-sorted data graphs for additional ratios. Graphs are attached for:
Medium14/Weak14Medium15/Positive15Medium15/Weak15Positive15/Weak15
(PDF)
