# Correction: Reproducibility of Fluorescent Expression from Engineered Biological Constructs in *E*. *coli*

**DOI:** 10.1371/journal.pone.0157255

**Published:** 2016-06-03

**Authors:** Jacob Beal, Traci Haddock-Angelli, Markus Gershater, Kim de Mora, Meagan Lizarazo, Jim Hollenhorst, Randy Rettberg, iGEM Interlab Study Contributors

The Acknowledgments contain several errors in the “2014 iGEM Interlab Study Contributors” section. In the “ETH_Zurich” subsection, the correct spelling of “Verena Jagger” is “Verena Jäggin”. In the “HUST-China” subsection, the correct spelling of “Yunjun Yang” is “Yunjun Yan”. In the “Leicester” subsection, the name “Ross Campbell” should be listed after “Richard Badge”. In the “Marburg” subsection, the name “Daniel F. Hurtgen” should be listed after “Matthias Franz”.
