# Supplementary material for: Reproducibility of Fluorescent Expression from Engineered Biological Constructs in E. coli
Source: PLoS One. 2016 Mar 3;11(3):e0150182. doi: 10.1371/journal.pone.0150182 (PMC4777433; doi:10.1371/journal.pone.0150182)
Supplement: S1 File — (PDF) [file pone.0150182.s001.pdf]

Reproducibility of Fluorescent Expression from Engineered Biological Constructs in *E. coli*
Supporting Information S1 File: DNA Constructs

Jacob Beal, Traci Haddock-Angelli, Markus Gershater, Kim de Mora, Meagan Lizarazo, Jim Hollenhorst, Randy Rettberg, with the iGEM Interlab Study Contributors

DNA constructs for the two studies are based on material made publicly available in the iGEM Registry of Standard Biological Parts (`http://parts.igem.org/`). Part and vector identifiers are references to constructs in the registry, and may be obtained there directly. The constructs used in the studies, illustrated in the main text in Figure 1 are listed in Table 1 (2014) and Table 2 (2015). Note that the controls in the 2015 study were recommended, not required, and as such are not present in all data sets.

All constructs studied are either a part directly available from the registry (in appropriate plasmid backbone) or else a BioBrick Standard Assembly [1] of two registry parts. Here we use the notation "Part1 + Part2" to mean a BioBrick assembly of two constructs, Part1 and Part2, that inserts Part1 into the plasmid containing Part2.

Two plasmid backbones are used in the study:

- The BBa_I20260 construct for the 2014 study uses vector pSB3K3, a low-to-medium copy plasmid with kanamycin resistance.
- All other constructs use vector pSB1C3, a high copy plasmid with chloramphenicol resistance.

Device	Construct
Strong14	BBa_J23101 + BBa_E0240 (B0032-E0040-B0015) in pSB1C3
Medium14	BBa_I20260 (J23101-B0032-E0040-B0015) in pSB3K3
Weak14	BBa_J23115 + BBa_E0240 (B0032-E0040-B0015) in pSB1C3

Table 1: Synthetic constitutive fluorescence constructs used for the 2014 iGEM Interlab Study. All numbers reference constructs publicly distributed from the iGEM Parts Repository (`http://parts.igem.org/`).

Device	Construct
Strong15	BBa_J23101 + BBa_I13504 (B0034-E0040-B0015) in pSB1C3
Medium15	BBa_J23106 + BBa_I13504 (B0034-E0040-B0015) in pSB1C3
Weak15	BBa_J23117 + BBa_I13504 (B0034-E0040-B0015) in pSB1C3
Positive15 (Control)	BBa_I20270 (J23151-B0032-E0040-B0015) in pSB1C3
Negative15 (Control)	BBa_R0040 in pSB1C3

Table 2: Synthetic constitutive fluorescence constructs used for the 2015 iGEM Interlab Study. All numbers reference constructs publicly distributed from the iGEM Parts Repository (`http://parts.igem.org/`).

References

[1] Shetty RP, Endy D, Knight Jr TF. Engineering BioBrick vectors from BioBrick parts. Journal of biological engineering. 2008;2(1):1–12.
