# Supplementary material for: Reproducibility of Fluorescent Expression from Engineered Biological Constructs in E. coli
Source: PLoS One. 2016 Mar 3;11(3):e0150182. doi: 10.1371/journal.pone.0150182 (PMC4777433; doi:10.1371/journal.pone.0150182)
Supplement: S2 File — (PDF) [file pone.0150182.s002.pdf]

2015 InterLab Protocol

Every team that participates in the 2015 iGEM InterLab study needs to fill in this form. Fill this in as best as you can. It's primarily based on E. coli work, but most questions can apply to other organisms as well. If you have any questions or problems filling in this form, please email us at measurement at igem dot org.

* Required

Team name *

InterLab Study wiki page *

Please provide the direct link to your team's wiki page for the InterLab study. As explained in the InterLab requirements, all teams must have a specific page for the InterLab study on their wiki.

Individuals responsible for conducting InterLab study *

Please list everyone involved with creating the devices, measuring them, and processing the data. Please indicate which role each person filled.

List anyone else who should be credited, e.g., in a publication based on this data

Chassis and Safety Information

What chassis did you use? *
Please note the cell type you used to conduct the InterLab Study. Be as specific as possible. Example: E. coli K-12 DH5-alpha (not just E. coli)

What Biosafety Level is your chassis (underline your answer)? *
Remember - BSL3 and 4 organisms are banned in iGEM. If you have questions about safety, please email safety at igem dot org!

- ○ BSL1
- ○ BSL2
- ○ I'm not sure

What PPE did you utilize during your experiments (from cloning through to measuring the devices)? *
Note any Personal Protective Equipment (PPE) used during this process. This could include gloves, lab coats, long pants, etc.

Information about Cloning

These are questions related to cloning your parts into the three (3) devices that you need to participate for the InterLab Study. This is not a protocol for cloning the devices. Each team is expected to clone these devices on their own.

What DNA assembly method did you use to create your devices (underline your answer)? *
If you used multiple methods, please select all that apply.

- ○ BioBricks
- ○ Gateway
- ○ Gibson
- ○ Golden Gate
- ○ MoClo
- ○ DNA Synthesis
- ○ Other:

How did you validate the final devices (underline your answer)? *
If you used multiple methods, please select all that apply.

- ○ DNA Sequencing
- ○ Restriction Mapping
- ○ Other:

Please tell us about any challenges or problems you encountered during the cloning process. *
Be as specific as possible. This will help us improve the InterLab study for next year. If you had no problems, note that as well.

Protocol - Growing Cells for Measurement

These are steps and questions related to growing up your cells to measure your finished devices.

Step 1: Streak out an agar plates with your organism containing the device and any control organisms you used *

Please check off each step that you followed by placing an “X” in front of the step. If you did anything differently or extra, please note that in the "Other" box.

- Streak out 1 plate per device and control
- Incubate plates overnight (18-20 hours or until individual colonies are clearly visible) at 37 C
- Note the time grown in hours below in "other"
- Other:

Please provide the positive control(s) you used. *

Note the organism, device, and/or plasmid used. We recommend BBa_I20270, a GFP expression device in the pSB1C3 backbone (chloramphenicol resistant).

Please provide the negative control(s) you used. *

Note the organism, device, and/or plasmid used. We recommend having one negative control where you test cells without any plasmid added and one negative control with an empty vector transformed (we recommend BBa_R0040, which is pTetR in pSB1C3).

What type of agar did you use for this step? *

For E. coli, we recommend LB Agar supplemented with the appropriate antibiotic. For antibiotic concentrations, please follow these guidelines: http://parts.igem.org/Help:Protocols/Antibiotic_Stocks

Step 2: Inoculate liquid culture with your experimental devices and controls. *

Please check off each culture that you setup by placing an “X” in front of it. If you did anything differently or extra, please note that in the "Other" box.

- Device 1: J23101+I13504
- Device 2: J23106+I13504
- Device 3: J23117+I13504
- Positive control (noted above)
- Negative control (noted above)
- Other:

What type of vessel or container did you use to grow your cells (underline your answer)? *

- Test tube
- 15mL conical tube

- ○ 96-well plate - round wells, flat bottom
- ○ 96-well plate - round wells, round bottom
- ○ 96-well plate - square wells, flat bottom
- ○ 96-well plate - square wells, round bottom
- ○ 96-deep well plate
- ○ Flask
- ○ Other:

Please provide any detailed information about your vessel / container below. *

For example: What dimensions were your test tubes? What volume was your conical tube or flask? If you used a flask, were they baffled or smooth?

If you used a test tube or conical tube, how were the tubes oriented in the incubator (underline your answer)? *

- ○ Upright
- ○ At an angle
- ○ Lying down
- ○ N/A - used a different vessel
- ○ Other:

What type of media did you use? *

For E. coli, we recommend Luria Broth supplemented with the appropriate antibiotic. For antibiotic concentrations, please follow these guidelines: http://parts.igem.org/Help:Protocols/Antibiotic_Stocks

What volume did you use to grow your cells? *

For test tubes, we recommend at least 3 mL of media. For 96-well plates, we recommend at least 150 uL of media.

Did you set up biological replicates in triplicate? *

Biological replicates are where different samples that are expected to be identical are measured. This informs you about the variability across your organisms that contain the same device. For example, if you are using E. coli, this would be done by measuring the fluorescence from three (3) different colonies containing the same device.

- ○ Yes
- ○ No

If you answered "No" to the previous question, state exactly how many biological replicates you ran for each construct.
Remember: one of the InterLab requirements is to measure your samples in (at least) biological triplicates.

Step 3: Incubate your liquid cultures. *
Please check off each step that you followed by placing an “X” in front of the step. If you did anything differently or extra, please note that in the "Other" box.

- ○ Temperature at 37 C (if different, note in "Other" below)
- ○ Shaking at 300 rpm (if different, note in "Other" below)
- ○ Incubate for 16-18 hours (if different, note in "Other" below)
- ○ Other:

Step 3: Continued
If you need more space to explain your growth conditions, please reply below. We would expect this for mammalian work or non-traditional bacterial chassis - but all teams are welcome to add more details.

Plate Reader Measurement

This is a general guide for setting up your cells for plate readings. These steps and questions are meant to provide a general protocol and we ask that you follow them to the best of your ability. If you follow a different protocol, please note it in the final question in this section. If you did not use a plate reader, please answer the first question and then you may skip this rest of this section.

Did you measure your cells with a plate reader? *

- ○ Yes
- ○ No

Step 1: Obtain initial OD600 measurement of your overnight cultures.

Please check off each step that you followed by placing an “X” in front of the step. If you did anything differently or extra, please note that in the "Other" box.

- ○ Set your instrument to read OD600
- ○ Setup a 96-well plate or cuvette with your cultures
- ○ Take the measurement and record it
- ○ Other:

Step 2: Dilute your samples to an OD600 of 0.5

Please check off each step that you followed by placing an “X” in front of the step. If you did anything differently or extra, please note that in the "Other" box.

- ○ Calculate the dilution required for each sample
- ○ Dilute each sample
- ○ Re-measure your sample on OD600
- ○ If your OD600 is within 5% of 0.5, proceed
- ○ If your OD600 is outside that range, recalculate your dilution and remeasure until it's within 5%
- ○ Other:

Step 3: Measure your samples

Please check off each step that you followed by placing an “X” in front of the step. If you did anything differently or extra, please note that in the "Other" box.

- ○ Set your instrument to measure GFP
- ○ Measure your cells
- ○ Other:

Alternate Protocol or Additional Details

Did you follow a different protocol? Please describe your protocol below. You may also add more details below if you followed our protocol.

Other Forms of Measurement

Protocol for non-plate readers

Did you measure the cells with a different piece of equipment? Please describe your protocol below with as much detail as possible.

Feedback

Please rate your experience with filling in this InterLab Protocol form (underline your answer). *

- ○ Very easy to fill in, no problems
- ○ Took a long time to fill out, but was easy to understand
- ○ Did not understand one or two questions
- ○ Did not understand an entire section
- ○ Very difficult to use; numerous problems
- ○ Other:

Please let us know any other thoughts or comments you have about the InterLab study experience.
