# Supplementary material for: Reproducibility of Fluorescent Expression from Engineered Biological Constructs in E. coli
Source: PLoS One. 2016 Mar 3;11(3):e0150182. doi: 10.1371/journal.pone.0150182 (PMC4777433; doi:10.1371/journal.pone.0150182)
Supplement: S3 File — (PDF) [file pone.0150182.s003.pdf]

2015 InterLab Worksheet

Every team that participates in the 2015 iGEM InterLab study needs to fill in this form.
IMPORTANT: If you measured your devices using multiple pieces of equipment, please fill out this form for each different type of measurement you obtained.
If you have any questions or problems filling in this form, please email us at measurement at igem dot org.
* Required

Team name *

InterLab Study wiki page *
Please provide the direct link to your team's wiki page for the InterLab study. As explained in the InterLab requirements, all teams must have a specific page for the InterLab study on their wiki.

Individuals responsible for conducting InterLab study *
Please list everyone involved with creating the devices, measuring them, and processing the data. Please indicate which role each person filled. List anyone else who should be credited, e.g., in a publication based on this data.

Date of InterLab Study *
Please note the date(s) that the measurement was obtained.

Did your team participate in the Extra Credit (underline your answer)? *
Details for the Extra Credit in 2015 can be found here:http://2015.igem.org/Tracks/Measurement/Interlab_study

- ○ Yes
- ○ No

Equipment Information

What type of incubator did you use to grow your cells? *
Please provide as much information as possible in terms of the equipment type, name, and model.

If known, what was your incubator's throw (shaking diameter)?
This information is often found with the manufacturer's specifications for the incubator.

What piece of equipment did you use to measure the devices? *
Please provide as much information as possible in terms of the equipment type, name, and model.

When was this equipment last calibrated? *

Who calibrated the equipment? *

What was the wavelength of light you used to excite the cells? *

What was the filter/channel you used to capture the light emission from the cells? *

What was the sampling frequency? *

If you have other information or details you wish to provide about your equipment, please tell us below.

Protocol

Did you fill in the InterLab Protocol provided by the Measurement committee (underline your answer)? *

The InterLab Protocol is provided here: http://2015.igem.org/Tracks/Measurement/InterLab_Protocol

- ○ Yes
- ○ No

If "no", please briefly indicate why you did not fill in the provided protocol.

All teams are required to fill this form in to the best of their ability.

Did you follow the InterLab Protocol provided by the Measurement committee (underline your answer)? *

The InterLab Protocol is provided here: http://2015.igem.org/Tracks/Measurement/InterLab_Protocol

- ○ Yes
- ○ No

If "no", please briefly indicate why you did not follow the provided protocol.

You should have informed and received approval from the Measurement Committee if you followed a different protocol.

How did you determine the final dataset that you are reporting? *

Please indicate how you processed your raw data in as much detail as possible. If you did not process your data and are instead reporting raw data, please indicate that as well.

Results

Please provide your final measurements for the three devices below. Please make sure you match up your measurements with the correct device. We are expecting numerical data for each input asking for Device measurement information. We are collecting the individual measurements for each biological replicate, as well as the mean and standard deviation you derived from these measurements. Please provide all five data points (3 measurements, mean, standard deviation) for each device below. Note: If you did the Extra Credit, please fill in the "Extra Credit" section on the next page.

Units Reported *

Please note the units that you are reporting all of your measurement data in below. For example, note if your data is in arbitrary units, units of fluorescein, molecules of equivalent fluorescein (MEFL), or any other type of unit.

Device 1: J23101 + I13504, biological replicate 1 *

Please provide the measurement information for the first replicate of Device 1 below.

Device 1: J23101 + I13504, biological replicate 2 *

Please provide the measurement information for the second replicate of Device 1 below.

Device 1: J23101 + I13504, biological replicate 3 *

Please provide the measurement information for the third replicate of Device 1 below.

Device 1: J23101 + I13504, mean across triplicates *

Please provide the measurement information for the mean of Device 1 below.

Device 1: J23101 + I13504, standard deviation *

Please provide the measurement information for the standard deviation of Device 1 below.

Device 2: J23106 + I13504, biological replicate 1 *

Please provide the measurement information for the first replicate of Device 2 below.

Device 2: J23106 + I13504, biological replicate 2 *

Please provide the measurement information for the second replicate of Device 2 below.

Device 2: J23106 + I13504, biological replicate 3 *

Please provide the measurement information for the third replicate of Device 2 below.

Device 2: J23106 + I13504, mean across triplicates *
Please provide the measurement information for the mean of Device 2 below.

Device 2: J23106 + I13504, standard deviation *
Please provide the measurement information for the standard deviation of Device 2 below.

Device 3: J23117 + I13504, biological replicate 1 *
Please provide the measurement information for the first replicate of Device 3 below.

Device 3: J23117 + I13504, biological replicate 2 *
Please provide the measurement information for the second replicate of Device 3 below.

Device 3: J23117 + I13504, biological replicate 3 *
Please provide the measurement information for the third replicate of Device 3 below.

Device 3: J23117 + I13504, mean across triplicates *
Please provide the measurement information for the mean of Device 3 below.

Device 3: J23117 + I13504, standard deviation *
Please provide the measurement information for the standard deviation of Device 3 below.

Extra Credit

If you have conducted the three (3) technical replicates for Extra Credit, please provide the measurements below for the second and third technical replicates (the first technical replicate should be recorded above in the Results section). We are expecting numerical data for each input asking for Device measurement information. If you did not do the Extra Credit, please skip this page.

Units Reported

Please note the units that you are reporting all of your measurement data in below. (Note: This should be the same unit as in Results; if not, please explain why.)

Device 1: J23101 + I13504, technical replicate 2, biological replicate 1

Please provide the measurement information for the indicated replicate of Device 1 below.

Device 1: J23101 + I13504, technical replicate 2, biological replicate 2

Please provide the measurement information for the indicated replicate of Device 1 below.

Device 1: J23101 + I13504, technical replicate 2, biological replicate 3

Please provide the measurement information for the indicated replicate of Device 1 below.

Device 1: J23101 + I13504, technical replicate 3, biological replicate 1

Please provide the measurement information for the indicated replicate of Device 1 below.

Device 1: J23101 + I13504, technical replicate 3, biological replicate 2

Please provide the measurement information for the indicated replicate of Device 1 below.

Device 1: J23101 + I13504, technical replicate 3, biological replicate 3

Please provide the measurement information for the indicated replicate of Device 1 below.

Device 1: J23101 + I13504, mean across technical replicates

Please provide the measurement information for the mean of Device 1 across the three technical replicates below.

Device 1: J23101 + I13504, standard deviation across technical replicates

Please provide the measurement information for the standard deviation of Device 1 across the three technical replicates below.

Device 2: J23106 + I13504, technical replicate 2, biological replicate 1

Please provide the measurement information for the indicated replicate of Device 2 below.

Device 2: J23106 + I13504, technical replicate 2, biological replicate 2
Please provide the measurement information for the indicated replicate of Device 2 below.

Device 2: J23106 + I13504, technical replicate 2, biological replicate 3
Please provide the measurement information for the indicated replicate of Device 2 below.

Device 2: J23106 + I13504, technical replicate 3, biological replicate 1
Please provide the measurement information for the indicated replicate of Device 2 below.

Device 2: J23106 + I13504, technical replicate 3, biological replicate 2
Please provide the measurement information for the indicated replicate of Device 2 below.

Device 2: J23106 + I13504, technical replicate 3, biological replicate 3
Please provide the measurement information for the indicated replicate of Device 2 below.

Device 2: J23106 + I13504, mean across technical replicates
Please provide the measurement information for the mean of Device 2 across the three technical replicates below.

Device 2: J23106 + I13504, standard deviation across technical replicates
Please provide the measurement information for the standard deviation of Device 2 across the three technical replicates below.

Device 3: J23117 + I13504, technical replicate 2, biological replicate 1
Please provide the measurement information for the indicated replicate of Device 3 below.

Device 3: J23117 + I13504, technical replicate 2, biological replicate 2
Please provide the measurement information for the indicated replicate of Device 3 below.

Device 3: J23117 + I13504, technical replicate 2, biological replicate 3
Please provide the measurement information for the indicated replicate of Device 3 below.

Device 3: J23117 + I13504, technical replicate 3, biological replicate 1
Please provide the measurement information for the indicated replicate of Device 3 below.

Device 3: J23117 + I13504, technical replicate 3, biological replicate 2
Please provide the measurement information for the indicated replicate of Device 3 below.

Device 3: J23117 + I13504, technical replicate 3, biological replicate 3
Please provide the measurement information for the indicated replicate of Device 3 below.

Device 3: J23117 + I13504, mean across technical replicates
Please provide the measurement information for the mean of Device 3 across the three technical replicates below.

Device 3: J23117 + I13504, standard deviation across technical replicates
Please provide the measurement information for the standard deviation of Device 3 across the three technical replicates below.

Feedback

Please rate your experience with conducting the InterLab Study (underline your answer) *

- ○ Very easy to participate; little to no problems
- ○ Cloning problems made it more difficult than expected
- ○ Equipment problems made it more difficult than expected
- ○ Instructions were unclear and made it difficult to participate
- ○ Very difficult to participate; numerous problems
- ○ Other:

Please rate your experience with filling in this InterLab Worksheet (underline your answer) *

- ○ Very easy to fill in, no problems
- ○ Took a long time to fill out, but was easy to understand
- ○ Did not understand one or two questions
- ○ Did not understand an entire section
- ○ Very difficult to use; numerous problems
- ○ Other:

Please let us know any other thoughts or comments you have about the InterLab study experience.
