# Supplementary material for: Reproducibility of Fluorescent Expression from Engineered Biological Constructs in E. coli
Source: PLoS One. 2016 Mar 3;11(3):e0150182. doi: 10.1371/journal.pone.0150182 (PMC4777433; doi:10.1371/journal.pone.0150182)
Supplement: S4 File — Document provided is a snapshot of the page as of October 4th, 2015, which includes information presented on participation and the preliminary results of the study. (PDF) [file pone.0150182.s004.pdf]

Tracks/Measurement/Interlab study

TRACKS:
ENERGY
ENVIRONMENT
FOOD AND NUTRITION
FOUNDATIONAL ADVANCE
HEALTH AND MEDICINE
INFORMATION PROCESSING
MANUFACTURING
NEW APPLICATION

NEW TRACKS:
ART AND DESIGN
COMMUNITY LABS
ENTREPRENEURSHIP
MEASUREMENT
Introduction
Track Details
Requirements
Interlab Study
Medal Criteria
Committee

iGEM 2014 Measurement Interlab Study

Introduction

All iGEM teams are invited and encouraged to participate in the first international interlab measurement study in synthetic biology. We're hoping this study will get you excited for iGEM and help prepare you for the summer!

Please note: All Measurement Track teams are **required** to participate in the interlab study.

The goal of the interlab study is to obtain fluorescence data for three specific genetic devices expressing GFP from iGEM teams around the world. Can you measure fluorescence somewhere in your lab? Then this is the perfect study for you! Even if your lab or the organisms you work with mean that you can't measure GFP from the specific devices, we want every team to be able to participate: email ***measurement at igem dot org*** and we will work out an alternative.

All teams who participate in the interlab study will be acknowledged at the Giant Jamboree with a special Measurement Prize!

For any questions, contact ***measurement at igem dot org*** .

Participating Teams

A big congratulations to the iGEM community for your enthusastic response! This year, 45 teams from all around the world have signed up to participate in the interlab study! The map below shows the locations of all of the teams participating in the interlab study --- some are pretty close together so you may need to zoom in to see them all!

Overall, the interlab study has been a big success, and the organizers want to thank everybody who has participated to make it such a a success. At the Jamboree, there will be two special events related to the interlab study:

- There will be a short workshop to discuss successes and challenges for both teams and organizers, with an eye toward making the study even bigger and better next year.

MICROFLUIDICS
POLICY AND PRACTICES
SOFTWARE

- The actual results of the interlab study will be presented to all of iGEM in a plenary session

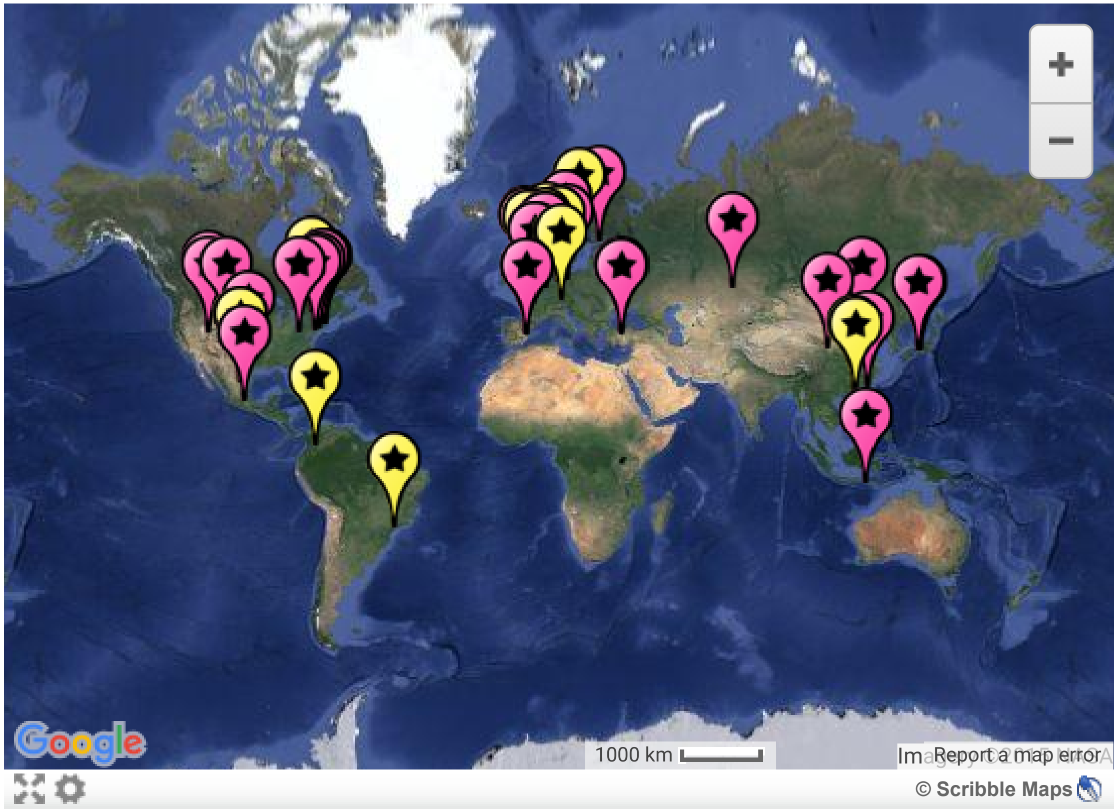

Registration and Timeline

Teams intending to participate in the Interlab study should sign up by emailing ***measurement at igem dot org*** .

Schedule:

- Sign-up Deadline: **September 1st, 2014**
- Data Due: **September 19th, 2014**
- Results Announced, Prizes Awarded: **At Giant Jamboree!**

Interlab Study Requirements

1. Each participating team must have an “Interlab Study” page on their wiki for easy reference for the Measurement Judges.
    1. All devices measured for this study should be listed on this page
    2. All protocols followed for this study should be linked/posted on this page

3. Sequencing data for all measured devices should be posted on this page
    1. **Note**: A restriction map (a restriction digest run on a gel) confirming the correct size of the device will suffice if DNA sequencing is not possible

2. Each team must use the three BioBrick devices listed below in the “Required Devices” section.
    1. **Note**: Teams are encouraged to measure more devices (see “Extra Credit” section below), but the three required devices must be included for the Interlab Study (unless an alternative set is required and negotiated via email to ***measurement at igem dot org*** .

3. Each participating team must collect and submit fluorescence data for these three devices (the data will be submitted through the “Interlab Study” form, see step 4).
    1. This data may be obtained by any means possible for the teams. Any instrument capable of measuring GFP is acceptable!
    2. Measurement data should be submitted in absolute units if possible. There are many ways this can be done, depending on your lab. If your lab cannot measure absolute units, relative units are acceptable.
        1. **Hint**: if you have access to a flow cytometer, absolute units can be measured by calibrating against standard fluorescent beads. A method for doing so is described (and supported with online tools) at: TASBE Tools.
    3. Data must be measured in triplicate (i.e., three samples for each device).
        1. **Note**: The mean and standard deviation across the replicates must be included.
        2. All teams must fill in the “Interlab Study” form where they will indicate the equipment used to measure the cells, describe how the data was collected, and list all controls used.

Required Devices

The three specific devices teams are required to measure fluorescence data for are given below.

Ideal culturing conditions for *E. coli*: Fluorescence should be measured after 16-18 hours of growth in a liquid culture (a simple overnight culture should work well). For *E. coli*, please grow the cultures in LB broth supplemented with the appropriate antibiotic. These should be grown at 37C with shaking (300 rpm, if possible). The strain of *E. coli* should be your normal cloning strain, ideally a DH5-alpha strain or similar (example: alpha-select cells from Bioline or Top10 cells from Invitrogen). The strain should be noted when sending in the data. **Non-*E. coli* Work:** For non-*E. coli* work, these conditions and/or devices may not work in your cells; however, we still want you to participate! Please contact us at ***measurement at igem dot org*** and let us know what cell type/cell line you plan to use, your measurement protocol, and the constructs you plan to measure to participate in the Interlab Study.

Important notes: The first device is already built and available in the distribution kit. The second and third devices must be built. You can do this using the BioBricks standard protocol by the teams participating in this study, or other methods if you prefer.

1. Existing device: BBa_I20260 (J23101-B0032-E0040-B0015) in the pSB3K3 vector.

        1. Kit location
            1. Plate 4, Well 18A

2. New device to be built by the iGEM team: BBa_J23101 + BBa_E0240 (B0032-E0040-B0015), must be built in the pSB1C3 backbone
    1. Kit locations
        1. BBa_J23101 (called BBa_K823005 when in pSB1C3): Plate 1, Well 20K
        2. BBa_E0240 (in pSB1C3): Plate 2, Well 24B

3. New device to be built by the iGEM team: BBa_J23115 + BBa_E0240 (B0032-E0040-B0015), must be built in the pSB1C3 backbone
    1. Kit locations
        1. BBa_J23115 (called BBa_K823012 when in pSB1C3): Plate 1, Well 22I
        2. BBa_E0240 (in pSB1C3): Plate 2, Well 24B

[redacted]*nd instead matches the K823012 sequence (which was already known to have these mismatches). You can either (a) use the J23115 part as distributed or (b) re-clone J23115 and correct the mismatch. Please let us know which path you will choose at* ***measurement at igem dot org****. Thank you!*

Extra Credit Opportunity for the Interlab Study

Have you finished your Interlab Study requirements above? Still eager for some more measurement fun? Then these “extra credit” assignments are right for you!

For teams looking to do some measurement work beyond the interlab study requirements, we are offering three chances for “extra credit”. You can complete any or all of these extra credit assignments if you complete the Interlab Study.

Teams who go above and beyond the interlab study will be acknowledged for each “extra credit” assignment completed at the Giant Jamboree with a special Measurement Prize! Each assignment is weighed equally.

Please note: Your team must complete the Interlab Study requirements listed above in order to earn any “extra credit”!

1. Measure cell-to-cell variation for the three required devices.
    1. Can you measure single cells? Or can you find a team that can help out? If so, then you can complete this “extra credit” assignment (and maybe squeeze in a collaboration between iGEM teams as well)!

2. Build and test at least three devices that are identical to devices that you have measured, except that they express RFP rather than GFP. Compare the fluorescence for RFP devices to the fluorescence for GFP devices.
    1. Are you interested in seeing how regulatory and protein elements interact? Some interactions are very sensitive and others more robust. Can you find a pattern of interaction? Absolute fluorescent

units can be very helpful here!

3. Build E0240 devices with the entire Anderson library of constitutive promoters (J23100-J23119) following standard BioBrick protocols and submit measurements for GFP expression for all devices.
    1. Are you interested in seeing how minor changes in promoter sequences can impact gene expression? If so, then this “extra credit” assignment is right for you! (Note: Measure this set of parts the same way you measured the required devices.)

For any questions, contact ***measurement at igem dot org*** .

GOOD LUCK!
