# Supplementary material for: Reproducibility of Fluorescent Expression from Engineered Biological Constructs in E. coli
Source: PLoS One. 2016 Mar 3;11(3):e0150182. doi: 10.1371/journal.pone.0150182 (PMC4777433; doi:10.1371/journal.pone.0150182)
Supplement: S5 File — Document provided is a snapshot of the page as of October 4th, 2015. (PDF) [file pone.0150182.s005.pdf]

Tracks/Measurement/Interlab study

iGEM 2015 Measurement Interlab Study

Late registration is open until June 30, 2015!

Introduction

All iGEM teams are invited and encouraged to participate in the Second International InterLab Measurement Study in synthetic biology. We're hoping this study will get you excited for iGEM and help prepare you for the summer!

Please note: All Measurement Track teams are **required** to participate in the InterLab study.

The goal of the InterLab study is to obtain fluorescence data for three specific genetic devices expressing GFP from iGEM teams around the world. Can you measure fluorescence somewhere in your lab? Then this is the perfect study for you! Even if your lab or the organisms you work with mean that you can't measure GFP from the specific devices, we want every team to be able to participate: email ***measurement at igem dot org*** and we will work out an alternative.

All teams who participate in the InterLab study will be acknowledged at the Giant Jamboree with a special Measurement Prize!

For any questions, contact ***measurement at igem dot org*** .

Registration and Timeline

Teams intending to participate in the InterLab study should sign up by emailing ***measurement at igem dot org*** .

Schedule:

- Sign-up Deadline: **June 19, 2015**
- Data Due: **August 28, 2015**
- Wiki Page: **At wiki freeze**
- Results Announced, Prizes Awarded: **At Giant Jamboree!**

InterLab Study Requirements

1. Each participating team must have an "InterLab Study" page on their wiki for easy reference for the Measurement Judges.
    1. All devices measured for this study should be listed on this page
    2. All protocols followed for this study should be linked/posted on this page

3. Sequencing data for all measured devices should be posted on this page
    1. **Note**: A restriction map (a restriction digest run on a gel) confirming the correct size of the device will suffice if DNA sequencing is not possible
2. Each team must use the three BioBrick devices listed below in the "Required Devices" section.
    1. **Note**: Teams are encouraged to measure more devices, but the three required devices must be included for the InterLab Study (unless an alternative set is required and negotiated via email to ***measurement at igem dot org***).
3. Each participating team must collect and submit fluorescence data for these three devices (the data will be submitted through the "InterLab Worksheet" form, see step 4).
    1. This data may be obtained by any means possible for the teams. Any instrument capable of measuring GFP is acceptable!
    2. Measurement data should be submitted in absolute units if possible. There are many ways this can be done, depending on your lab. If your lab cannot measure absolute units, relative units are acceptable.
        1. **Hint**: If you have access to a flow cytometer, absolute units can be measured by calibrating against standard fluorescent beads. A method for doing so is described (and supported with online tools) at: **TASBE Tools**
        1. **Previous teams who have generated absolute units:** For flow cytometry measurements, we know of two previous teams: **BostonU 2013** and **BostonU 2014**. For plate reader measurements, we know of one previous team: **Imperial 2014**. (If you know of more teams that should be included, please email us at ***measurement at igem dot org*** and we'll be happy to add them!)
    3. Data must be measured in *triplicate*. In other words, we expect each participating team to provide us with three (3) **biological** replicates for each device. **Biological replicates** are where different samples that are expected to be identical are measured. This informs you about the variability across your organisms that contain the same device. For example, if you are using *E. coli*, this would be done by measuring the fluorescence from three (3) different colonies containing the same device.
        1. **Note:** The mean and standard deviation across the replicates must be included.
4. All teams must fill in the **InterLab Worksheet** where they will **(a)** report their results, **(b)** indicate the equipment used to measure the cells, and **(c)** list all controls used.
5. All teams must follow and fill in the **InterLab Protocol** to the best of their ability. This protocol is based on using *E. coli* and provides a basic protocol for students to follow for **(a)** culturing their cells and **(b)** obtaining plate reader measurements. If you used a different chassis, please fill the form in as much as possible.
    1. **Note:** If you don't have a plate reader and/or plan to collect the data using a different piece of equipment, we still want you to participate! Please email us at ***measurement at igem dot org*** and let us know what type of measurements you plan on obtaining.

Required Devices

The three specific devices teams are required to measure fluorescence data for are given below.

Important notes: The three devices you need to measure must be built by your team. You can do this using the BioBricks standard protocol or other methods if you prefer, including synthesis. You must note how you made your devices when you submit your results. Also, the GFP Generator (BBa_I13504) listed below is in the pSB1A2 backbone to make assembly easier if you follow the BioBricks

standard protocol. This part also exists in the pSB1C3 backbone in the distribution. We recommend you use the one listed below to make antibiotic selection easier during assembly.

1. Device 1: J23101 + I13504 (B0034-E0040-B0015), must be built in the pSB1C3 backbone
    1. Kit locations
        1. J23101 (called K823005 when in pSB1C3): 2015 Kit Plate 1, Well 20K, pSB1C3
        2. I13504: 2015 Kit Plate 4, Well 21J, pSB1A2
2. Device 2: J23106 + I13504 (B0034-E0040-B0015), must be built in the pSB1C3 backbone
    1. Kit locations
        1. J23106 (called K823008 when in pSB1C3): 2015 Kit Plate 1, Well 22A, pSB1C3
        2. I13504: 2015 Kit Plate 4, Well 21J, pSB1A2
3. Device 3: J23117 + I13504 (B0034-E0040-B0015), must be built in the pSB1C3 backbone
    1. Kit locations
        1. J23117 (called K823013 when in pSB1C3): 2015 Kit Plate 1, Well 22K, pSB1C3
        2. I13504: 2015 Kit Plate 4, Well 21J, pSB1A2

Thank you: Many thanks to the Carnegie Mellon team for helping make the InterLab compatible with BioBricks assembly this year! Due to human error, the original devices used promoters that were not BioBricks compatible. The updated devices (shown above) are using J23101, J23106, and J23117 which are all BioBricks compatible in the Distribution Kit for the 2015 season.

Chassis Usage

E. coli: When using *E. coli* as your chassis, please indicate the strain you are using when you fill out the InterLab Study Form. Ideally, we want to know as much about your strain as possible. For example, please note if you are using a common cloning strain such as DH5-alpha or TOP10 cells.

Non-*E. coli* Work: For non-*E. coli* work, these devices may not work in your cells; however, we still want you to participate! Please contact us at ***measurement at igem dot org*** and let us know what cell type/cell line you plan to use, your measurement protocol, and the constructs you plan to measure to participate in the InterLab Study.

Extra Credit Opportunity for the InterLab Study

Have you finished your InterLab Study requirements above? Still eager for some more measurement fun? Then this “extra credit” assignment is right for you!

For teams looking to do some measurement work beyond the InterLab study requirements, we are offering one chance for “extra credit”.

Teams who go above and beyond the InterLab study will be acknowledged for their work at the Giant Jamboree!

Please note: Your team must complete the InterLab Study requirements listed above in order to earn any “extra credit”!

Extra Credit for 2015: This year, we are asking teams to provide data from at least three (3) **technical replicates** of their experiment for extra credit. Technical replicates are defined as replicates that share the same sample, where the measurements are repeated. In this case, we are asking teams to measure their experimental samples at least three (3) times to establish the variability of your chosen analysis technique (plate reader, flow cytometry, etc.).

As mentioned above, this is different from **biological replicates**, which is where different samples that are expected to be identical are measured. For example, if you are using *E. coli*, then you would select three different colonies containing your device to grow up in triplicate to measure the variability between organisms that should be identical. In this example, you are creating three (3) **biological** replicates.

For any questions, contact ***measurement at igem dot org*** .

GOOD LUCK!

InterLab Results from 2014

In 2014, 45 teams representing 18 countries from all around the world signed up to participate in the InterLab study! We're hoping to make the 2015 InterLab study even bigger this year and we can only do that with **your** help. The map below shows the locations of all of the teams participating in the interlab study - some are pretty close together so you may need to zoom in to see them all! Pink locations indicate teams who participated in the InterLab study while Yellow locations indicate teams who participated in both the InterLab study and the Extra Credit.

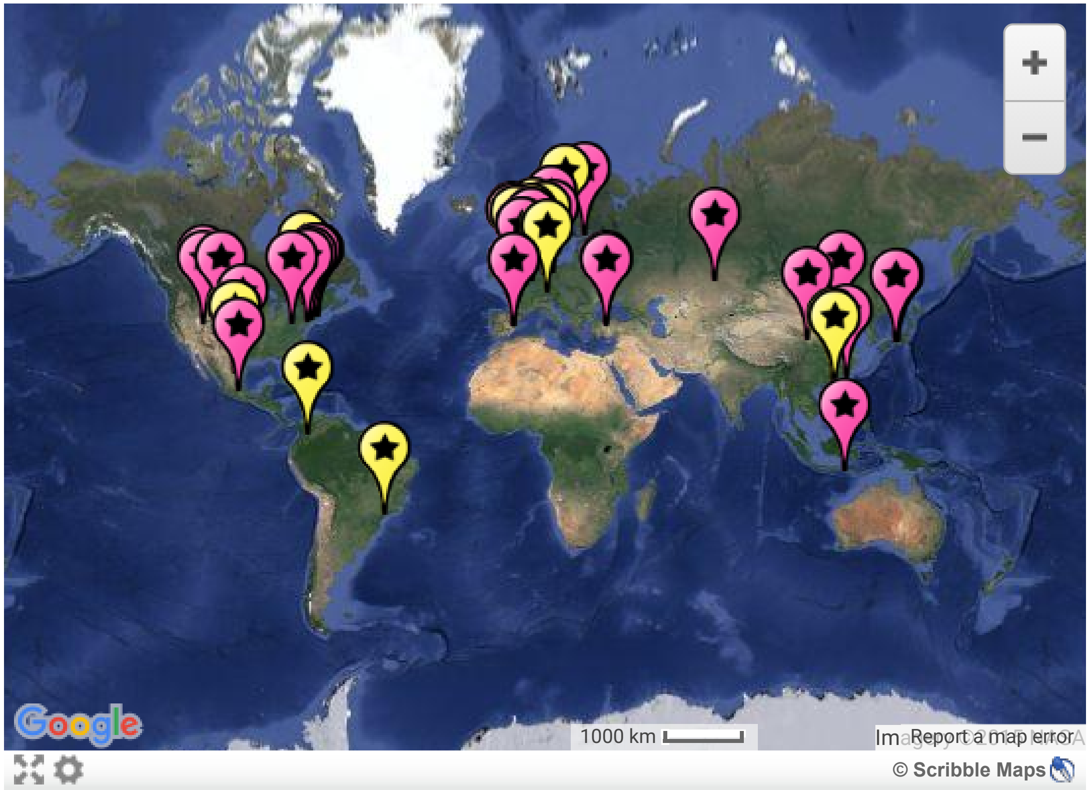

During the Awards Ceremony at the 2014 Giant Jamboree, Dr. Jacob Beal, the Chair of the Measurement Track, presented the preliminary results from the 2014 InterLab study. It was the largest InterLab study ever conducted in Synthetic Biology, with 37 out of the 45 teams sending in datasets along with the protocols used to collect that data. Please check out the video below for Jake's presentation from the Giant Jamboree!

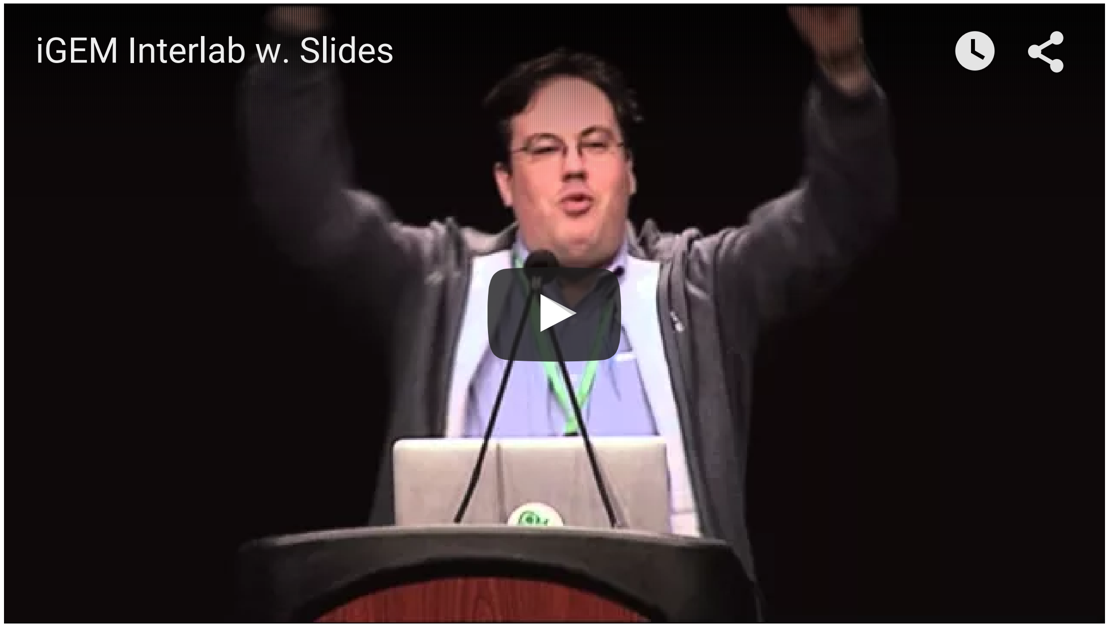
iGEM Interlab w. Slides
