# Supplementary figures and images for: Reproducibility of Fluorescent Expression from Engineered Biological Constructs in E. coli
Source: PLoS One. 2016 Mar 3;11(3):e0150182. doi: 10.1371/journal.pone.0150182 (PMC4777433; doi:10.1371/journal.pone.0150182)

# S10 Additional Log-Normal Distribution Graphs

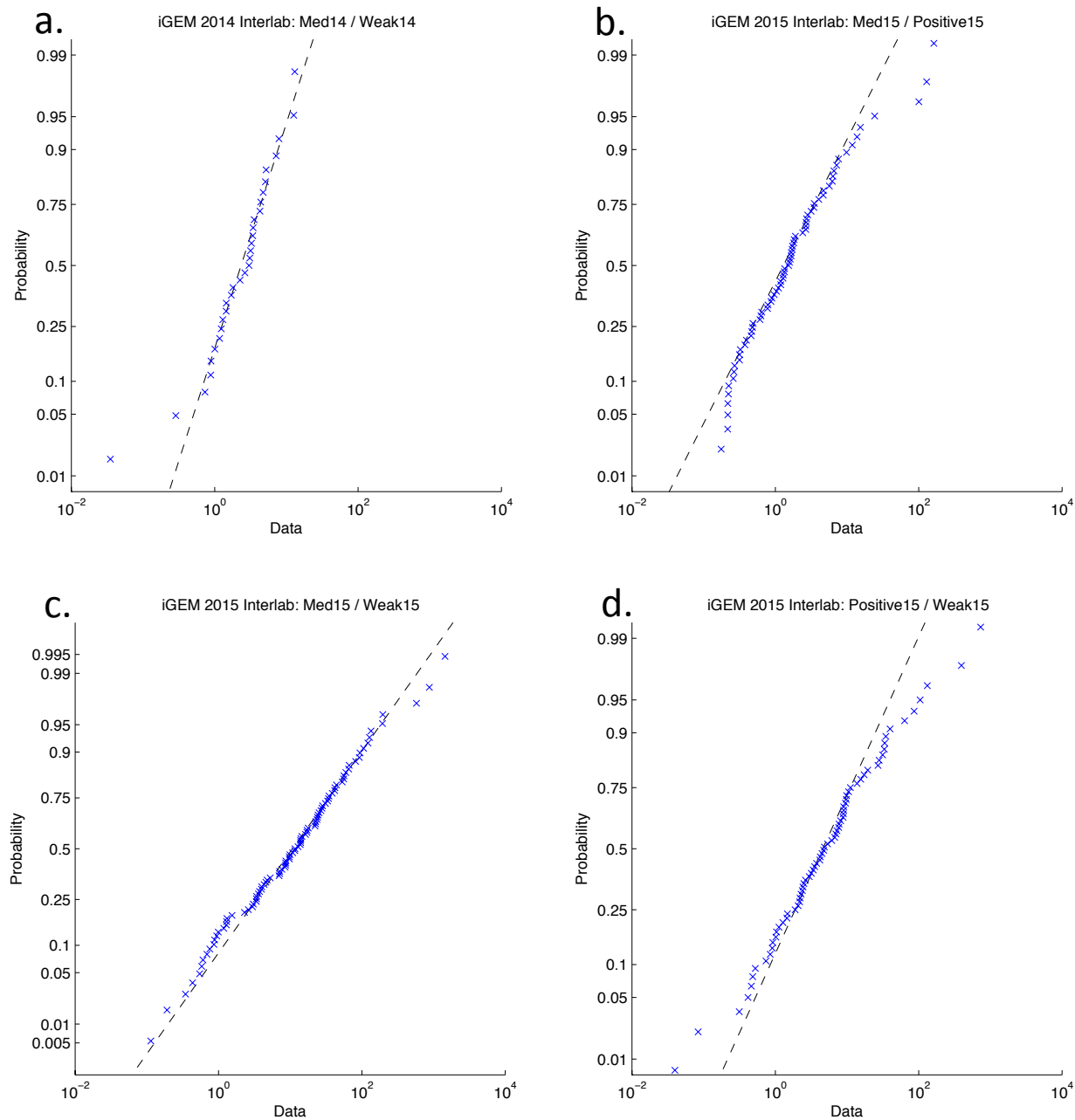

Supplement: S1 Fig — Graphs are attached for: Medium14/Weak14Medium15/Positive15Medium15/Weak15Positive15/Weak15 (PDF) [file pone.0150182.s010.pdf]

# S11 Additional Rank-Sorted Data Graphs

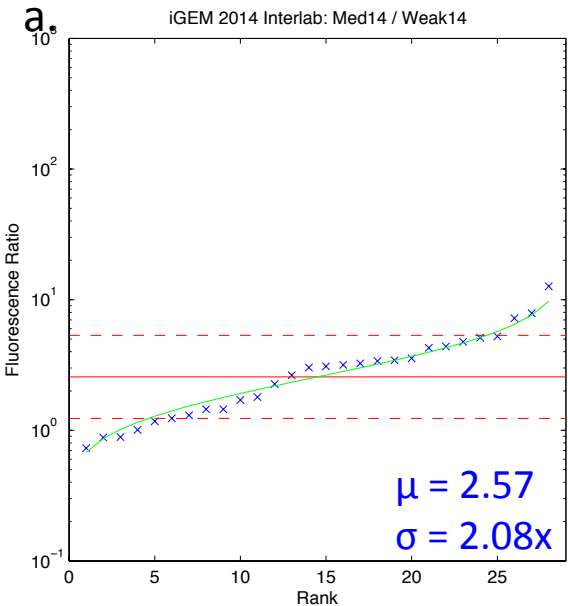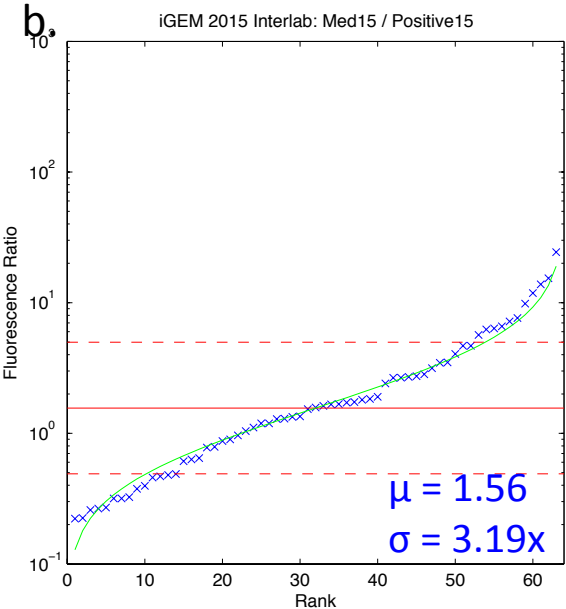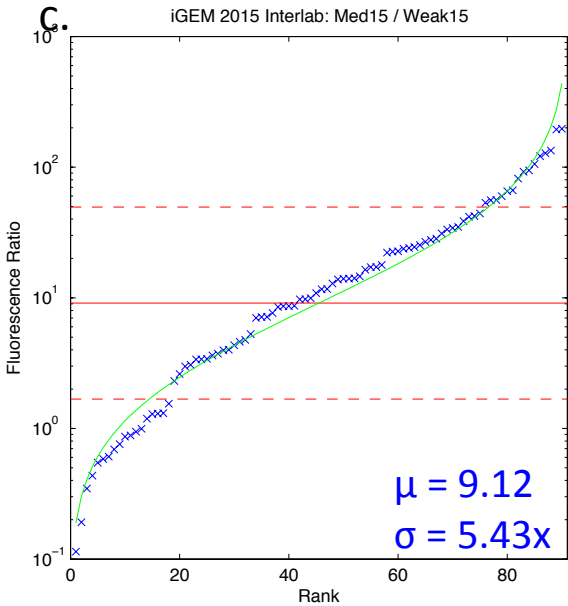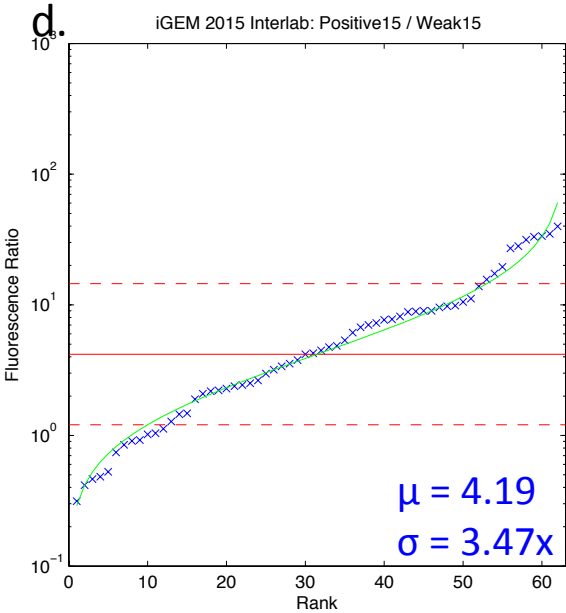

Supplement: S2 Fig — Graphs are attached for: Medium14/Weak14Medium15/Positive15Medium15/Weak15Positive15/Weak15 (PDF) [file pone.0150182.s011.pdf]
